# Directed evolution engineering to improve activity of glucose dehydrogenase by increasing pocket hydrophobicity

**DOI:** 10.3389/fmicb.2022.1044226

**Published:** 2022-11-09

**Authors:** Li Hua, Bao Qianqian, Zhao Jianfeng, Xu Yinbiao, Yang Shengyu, Xue Weishi, Sun Yang, Liu Yupeng

**Affiliations:** ^1^School of Life Sciences, Institute of Microbial Engineering, Henan University, Kaifeng, China; ^2^Engineering Research Center for Applied Microbiology of Henan Province, Henan University, Kaifeng, China

**Keywords:** glucose dehydrogenase, AlphaFold2, site-directed mutagenesis, hydrophobicity, organic solvents

## Abstract

Glucose dehydrogenase (GDH) is a NAD(P)^+^ dependent oxidoreductase, which is useful in glucose determination kits, glucose biosensors, cofactor regeneration, and biofuel cells. However, the low efficiency of the catalysis hinders the use of GDH in industrial applications. In this study, an analysis of interactions between eight GDH mutants and NADP^+^ is powered by AlphaFold2 and Discovery Studio 3.0. The docking results showed that more hydrogen bonds formed between mutants, such as P45A and NADP^+^, which indicated that these mutants had the potential for high catalytic efficiency. Subsequently, we verified all the mutants by site-directed mutagenesis. It was notable that the enzyme activity of mutant P45A was 1829 U/mg, an improvement of 28-fold compared to wild-type GDH. We predicted the hydrophobicity of the protein-ligand complexes, which was confirmed by an 8-anilino-1-naphthalenesulphonic acid fluorescent probe. The following order of increasing hydrophobicity index was deduced: GDH < N46E < F155Y < P45A, which suggested that the enzyme activity of GDH is positively related to its pocket hydrophobicity. Furthermore, P45A still showed better catalytic ability in organic solvents, reaching 692 U/mg in 10% isopropanol, which was 19-fold that of the wild-type GDH. However, its substrate affinity was affected by organic solvents. This study provides a good theoretical foundation for further improving the catalytic efficiency of GDH.

## Introduction

Oxidoreductases are increasingly used as a powerful tool for catalyzing redox reactions due to their high degree of selective specificity, catalytic activity, and sustainable development, which are commonly used in the production of high-value drugs and polymer materials (Alpdagtas et al., 2018; Sheldon and Woodley, 2018). Glucose dehydrogenase (GDH, EC 1.1.1.47) is an oxidoreductase that is an important enzyme in the food and pharmaceutical industries and useful in glucose determination kits, glucose biosensors, cofactor regeneration, bioelectrodes, and biofuel cells (Xu et al., 2007; Wu et al., 2013; Stolarczyk et al., 2020). Its normally active protein is a tetramer, which is present in numerous microorganisms and the liver of animals (Kallberg et al., 2002).

At present, various NAD(P)^+^-dependent GDH have been isolated from several *Bacillus* species, including *Bacillus amyloliquefaciens* (Pongtharangkul et al., 2015), *Bacillus megaterium* (Xu et al., 2007), *Bacillus subtilis* (Strauss, 1983) and *Bacillus thuringiensis* (Vazquez-Figueroa et al., 2007). It was notable that these GDHs from *Bacillus* species have over 80% homology by amino acid sequence alignment, which are similar to other short-chain dehydrogenases in their overall folding and subunit architecture (Xu et al., 2007). In previous studies, the thermal stability of GDH was the focus of research. Baik et al. (2005) found that Q252L and E170K could synergistically stabilize the tetramer structure by strengthening the hydrophobic interactions at the dimer-dimer interface. Eduardo et al. used overlap extension to obtain the single variants F155Y, E170 (K or R), and Q252L with higher thermal stability. The half-life of the best variant was ~3.5 days at 65°C, with specific activities ranging from 100 to 145 U/mg (Vazquez-Figueroa et al., 2007). It was reported that the GDH mutant F20 from *B. megaterium* IWG3 was subjected to directed evolution by the family shuffling method and a highly thermostable mutant, GDH DN-46, was isolated with a half-life of 540 min at 66°C (Baik et al., 2003). Liang et al. (2013) constructed three mutants, namely Q252L/E170R/V149K, Q252L/E170R/G259A, and Q252L/E170R/V149K/G259A, which showed excellent thermostability. Among them, the half-life of mutant Q252L/E170R/V149K/G259A reached 3.8 days at 70°C. Even though mutants with high thermal stability were constructed, the issue of low enzyme activity was ignored, which hampered the use of GDH in industrial applications. In addition, the characterization of GDH in organic solvents, which is widely used for increasing substrate solubility, altering reaction thermodynamics, and discovering novel reactions (Serdakowski and Dordick, 2008; Doukyu and Ogino, 2010), is rarely reported.

In recent years, the redesign of oxidoreductases by protein engineering based on rational design or random mutations was used to improve their catalytic performance (Wang and Yiu, 2016). Aslan et al. constructed six variant libraries using a semi-rational design to expand the substrate selectivity of L-lactate dehydrogenase from the thermophilic organism *Geobacillus stearothermophilus* and achieved variants which could efficiently catalyze α-ketoacids for the preparation of corresponding α-hydroxy acids (Aslan et al., 2016). Berin Yelmazer et al. introduced mutations into the active sites of FDH in *Candida methylica* and *Chaetomium thermophilum* to explore the mechanism of affecting the binding of HCO_3_^−^ and driving hydrides away from the reaction in the opposite direction by specific mutations (Pala et al., 2018). In previous study, V144L variant of phenylalanine dehydrogenase (PheDH) from *Bacillus badius* was performed by site-directed mutation, whose substrate phenylalanine specificity was significantly increased by 4-fold comparing to the wild type (Yousefi et al., 2017). In the NADPH-dependent malate dehydrogenase (ScMDH) from *Streptomyces coelicolor*, four amino acid residues (Glu42, Ile43, Pro45, and Ala46) in the Rossmann fold region were set as target for mutation, which resulted in a 2231.3-fold shifted toward NADPH in the coenzyme specificity of ScMDH-T4 (Ge et al., 2014).

Previous studies found that GDH can catalyze either NAD^+^ or NADP^+^, whereas the *K*_cat_/*K*_m_ value for NADP^+^ was approximately 4-fold higher than that for NAD^+^ (Xuejiao, 2011). In this study, we describe the evolution of GDH from *B. subtilis* by site-directed mutagenesis and the characterization of GDH during the catalytic process through (1) analysis of the interaction between GDH mutants and NADP^+^ by AlphaFold2 and Discovery Studio 3.0, (2) comparison of the bioactivity of glucose dehydrogenase mutants using pET-28a(+) in the *Escherichia coli* expression system, (3) hydrophobic analysis of GDH mutants using 8-aniline-1-naphthalenesulfonic acid (ANS) fluorescent probe, SDS binding method, and curcumin, (4) characterization of GDH mutants purified *via* Ni^2+^ affinity chromatography, and (5) analysis of the catalytic activity and stability of GDH mutants affected by organic solvents. This study provides a good foundation for GDH research in the fields of bioanalysis, bioindustry, and bioenergy.

## Materials and methods

### Microorganisms and cultivation conditions

Mutations were generated with polymerase chain reaction (PCR), using pET-28a(+) plasmid containing the GDH gene as the template. The primers were shown in Table 1. *Bacillus subtilis* 163 was used for amplifying the gene encoding GDH by PCR. The seed cultures of *E. coli* Bl-21 and *B. subtilis* 163 were cultured in 20 ml test tubes containing 2 ml of lysogeny broth (LB) at 37°C and 200 rpm for 12 h. Kanamycin (50 μg/ml) was added to the growth media of recombinant *E. coli* Bl-21. LB medium contained peptone (10 g/L), yeast extract (5 g/L), and NaCl (10 g/L).

**Table 1:** Primers used in the experiment.

Primers	Primer sequences
GDH-F	ATGGGTCGCGGATCCGAATTCATGTATCCGGATTTAAAAGGAAAAG
GDH-R	TGCTCGAGTGCGGCCGCAAGCTTTTAACCGCGGCCTGCCTG
P45A-F	CAAGAT **G** **CG**AACGAGGTAAAAGAAGAGGTCATC
P45A-R	CTTTTACCTCGTT**CGC**ATCTTGTTTATTACTA
N46E-F	GATCCG **GAA**GAGGTAAAAGAAGAGGTCATCA
N46E-R	CTTCTTTTACCTC **TTC**CGGATCTTGTTTATTAC
F155Y-F	CCGTTA**TAC**GTCCACTATGCGGCAAGTAAAGGCG
F155Y-R	GCATAGTGGAC **GT** **A**TAACGGCCAAGGAATCAC
E170R-F	GATGACA **AG** **A**ACATTAGCGTTGGAATACGCGCCG
E170R-R	ACGCTAATGT**TCT**TGTCATCAGCTTTATCCCGCC
V227A-F	TCGCCGCA**GCT**GCAGCCTGGCTTGCTTCGAAG
V227A-R	AAGCCAGGCTGC **AG** **C**TGCGGCGATCTCCTCCGGT
W230F-F	GCAGCC**TTC**CTTGCTTCGAAGGAAGCCAGCTA
W230F-R	TTCGAAGCAAG **GA** **A**GGCTGCTACTGCGGCGATC
Q252L-R	ACGCTAATGT **TCT** TGTCATCAGCTTTATCCCGCC

Underlined text indicates mutant bases, while bold text indicates mutated amino acid sites.

### Analysis of the interaction between GDH and NADP^+^ by discovery studio 3.0

A three-dimensional (3D) model of GDH mutants was obtained using AlphaFold2 (Jumper et al., 2021). The PDB structure of NADP^+^ was retrieved from PubChem Compound of the NCBI GenBank database. Receptor-ligand interaction studies between GDH and NADP^+^ were carried out using Discovery Studio 3.0 (Zhang et al., 2021).

### Construction of plasmids and recombinant strains

All the primers for constructing plasmids are listed in Table 1. The PCR product of GDH was digested with *Eco*RI and *Hin*dIII and cloned into *Eco*RI-*Hin*dIII-digested pET-28a(+) after agarose gel purification. The ligation mixture was first transformed into chemically competent *E. coli* DH5α cells. The positive recombinants were further confirmed by PCR. The sequenced plasmid was transferred into competent *E. coli* Bl-21 cells. GDH mutants were constructed using the overlapping primer method (Sudhir et al., 2016). The plasmids were constructed using a FastPure Plasmid Mini Kit, FastPure Gel DNA Extraction Mini Kit, and ClonExpress II One Step Cloning Kit (Vazyme, Nanjing, China). Primer synthesis and nucleic acid sequencing were completed by Sangon Biotech (Zhengzhou, China).

### Expression and purification of GDH mutants

Recombinant *E. coli* Bl-21 was inoculated into 50 ml TB media with 50 μg/ml kanamycin, cultured at 37°C and 200 rpm for 2 h. Subsequently, 0.5 mM IPTG was added to the medium. After 12 h at 22°C and 200 rpm, cells were harvested by centrifugation at 12000 g, 4°C for 5 min, and washed three times with pH 7.5 phosphate buffer. The cells were ultrasonically broken and centrifuged at 4°C and 12,000 g for 20 min to obtain the crude enzyme solution. Protein purification was accomplished by Ni^2+^ affinity chromatography (Li et al., 2021). Finally, imidazole was removed by ultrafiltration. Protein was analyzed by SDS polyacrylamide gel electrophoresis (SDS-PAGE) using a 12% polyacrylamide gel. TB medium was composed of peptone (12 g/L), yeast extract (24 g/L), K_2_HPO_4_•3H_2_O (12.55 g/L), KH_2_PO_4_ (2 g/L), and glycerol (10 g/L). The protein content was quantified by the Bradford assay (Xu et al., 2007) with bovine serum albumin as standard.

### Enzyme assays

The GDH activity was assayed at 60°C for 5 min, using an incubation mixture containing 0.2 M NH_4_Cl (pH 9.0), 50 g/L NADP^+^, 1 M glucose, and 0.5 g/L GDH. All experiments were performed in a final volume of 4 ml and the amount of NADPH generated in the reaction was measured at 340 nm. One unit of enzymatic activity was defined as the amount of enzyme required for generating 1 μmoL of NADP^+^ per minute under the assay conditions.

### Characterization of GDH mutants

The enzyme activity was detected at different temperatures (25–80°C) to determine the optimal temperature of GDH, and then the optimal pH of GDH was determined from pH 3 to 12 at the optimal temperature. To analyze thermal stability, GDH mutants were treated at various temperatures (30°C–80°C) for 20 min. To determine pH stability, GDH mutants were kept in various pH buffers for 20 min. The untreated enzyme activity was taken as 100%. In addition, the half-life (*t*_1/2_) parameter was used to evaluate the thermal stability of GDH mutants (Zhou and Wang, 2021).

Kinetic studies were performed at optimum temperature and pH conditions. The kinetic parameters *K*_m_ and *V*_max_ were analyzed by a Lineweaver–Burk plot (double reciprocal plot), which uses 1/[S] as the *X* axis and 1/*V* as the *Y* axis (1) (Feng et al., 2018).


(1) $$\frac{1}{v}=\frac{K_{m}}{V_{\text{max}}}•\frac{1}{\left[S\right]}+\frac{1}{V_{\text{max}}}$$

### Determination of GDH hydrophobicity

To determine the hydrophobicity of GDH, the ANS fluorescent probe method, SDS binding method, and curcumin were used in this study (Cardamone and Puri, 1992; Kato et al., 2002; Noh et al., 2006; Sneharani, 2016; Kamshad et al., 2019). ANS and curcumin were purchased from Macklin Biochemical Co., Ltd. (Shanghai, China). GDH hydrophobicity was assayed at 30°C, using a sample containing 20 mmol/L Tris–HCl buffer (pH 7.4), 500 mmol/L NaCl, and 0.6 g/L protein. Subsequently, 20 μl ANS (8 mmol/L) was added to 1 ml of the aforementioned sample. The mix was left in the dark for 30 min. The fluorescence intensity (*λ*_ex_ 390 nm and *λ*_em_ 504 nm) of the samples was monitored at 30°C after shocking. The concentration of pure protein ranged from 0 to 5 g/L. The curve slope represents the protein hydrophobicity index (*S*_0_). For SDS binding and curcumin method, the absorbance of base blue SDS-methylene and fluorescence intensity of riboflavin were detected, which reflects the number of hydrophobic groups on the protein surface.

### Determination of stability and activity of GDH mutants in organic solvents

The parameters of enzymes were detected in a solution containing 10% (V/V) organic solvents (isopropanol, acetone, methanol, glycol, and DMSO; Munawar and Engel, 2013). Purified GDH mutants (0.5 g/L) were kept in a solution containing 10% (V/V) organic solvents for 10 min at room temperature for detecting organic solvent half-life (*t*_1/2_; Qian et al., 2020).

## Results and discussions

### Analysis of the interaction between GDH mutants and NADP^+^

**Figure 1:**
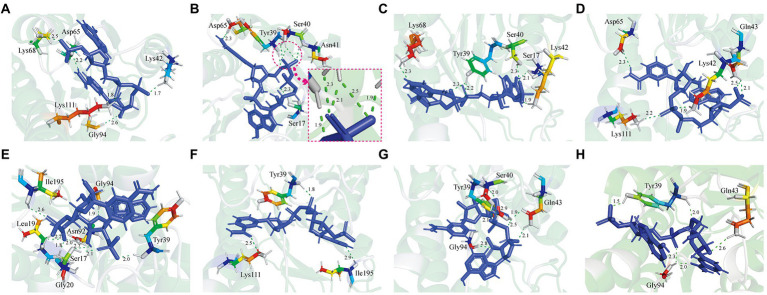
Analysis of the interaction between Glucose dehydrogenase (GDH) mutants and NADP^+^. **(A)** GDH-NADP^+^ complex. **(B)** P45A-NADP^+^ complex. **(C)** Q252L-NADP^+^ complex. **(D)** F155Y-NADP^+^ complex. **(E)** W230F-NADP^+^ complex. **(F)** N46E-NADP^+^ complex. **(G)** V227A-NADP^+^ complex. **(H)** E170R-NADP^+^ complex. The protein-ligand interactions between GDH mutants and NADP^+^ were studied using Discovery Studio 3.0. The green dashed line represents the hydrogen bonds. The unit of bond length is angstrom (Å).

In previous studies, it was suggested that several amino acid residues would influence the stability of GDH from *B. subtilis* 163 by significantly affecting the interactions between its subunits (Vazquez-Figueroa et al., 2007). However, the interactions between protein and ligand have not been comprehensively reported. In this research, we explored the binding between GDH mutants and ligand NADP^+^ due to its higher affinity than NAD^+^ (Xuejiao, 2011; Munawar and Engel, 2012; Stolarczyk et al., 2020). Above all, the structure prediction of eight GDH mutants was powered by AlphaFold2 (Supplementary Figures 1–8), which was used as a receptor for docking. The glucose dehydrogenase with NADP^+^ as substrate (PDB: 7V0H) from *Burkholderia cenocepacia* was used as a template. Subsequently, Discovery Studio 3.0 was used to analyze the protein-ligand interactions between GDH mutants and NADP^+^ (Figure 1). The docking parameters of all complexes are shown in Supplementary Tables 1–8. We found that a 1.7 Å hydrogen bond was formed between the amino group of Lys42 and hydroxyl oxygen of ligand NADP^+^ in the GDH-NADP^+^ complex (Figure 1A). In the P45A-NADP^+^ complex, there were another five hydrogen bonds (1.9–2.5 Å) formed with the phosphate group of ligand NADP^+^. This may be due to that the location of phosphate group affects the formation of hydrogen bond (Figure 1B). Among these, a 1.9 Å hydrogen bond occurred between the amino group of Tyr39 and the methyl oxygen of NADP^+^. Notably, the amino group of Ser40 in P45A can simultaneously form two hydrogen bonds with O-15 (2.5 Å) and O-16 (2.1 Å) of NADP^+^. In addition, the hydroxyl group of Ser40 also forms a hydrogen bond (2.3 Å) with O-16 of NADP^+^. Meanwhile, it was found that Lys42 was replaced by Ans41 in this complex compared with wild GDH, which can form a hydrogen bond with O-15 of NADP^+^. This result indicated that P45A may have a higher affinity for NADP^+^ than wild-type GDH. Similar amino acid residues occur between the P45A-NADP^+^ complex and the Q252L-NADP^+^ complex, such as Ser17, Tyr39, and Ser40 (Figure 1C). In the F155Y-NADP^+^ complex, the amino group of Gln43 could form 2 hydrogen bonds with O-39 (2.1 Å) and O-40 (2.5 Å), which is different from wild-type GDH (Figure 1D). In the other four mutants, different types of hydrogen bonds could be formed by Ser17, Leu19, Tyr39, Gln43, Gly20, Asn92, and Ile195 (Figures 1E–H). The docking results indicated that these mutants have the potential for high catalytic efficiency. Subsequently, site-directed mutagenesis was used to further evaluate their catalytic ability.

### Comparison of the bioactivity of glucose dehydrogenase mutants

**Figure 2:**
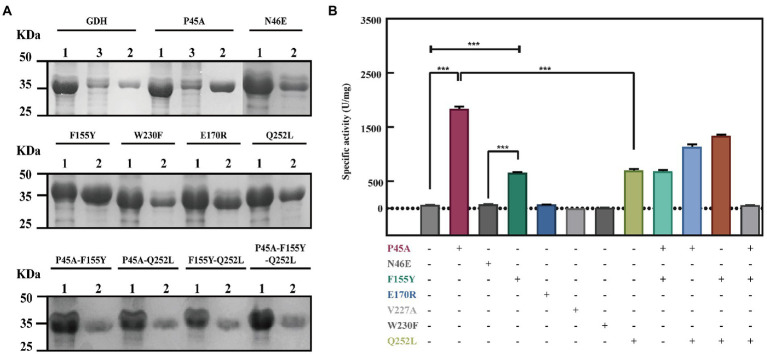
Improvement of the GDH catalytic ability by site-directed mutagenesis. **(A)** SDS polyacrylamide gel electrophoresis (SDS-PAGE) analysis of GDH mutants. Lane 1: crude enzyme solution; Lane 2: pure GDH mutants by Ni-NTA chromatography; Lane 3 flow-through of sample. 10 μg protein was analyzed by SDS-PAGE using 12% polyacrylamide gel. Protein bands were stained by Coomassie Brilliant Blue R250. **(B)** Evaluation of the mutants by site-directed mutagenesis. The results are expressed as the mean ± SD (n = 3). * Indicates *p* < 0.05, ** indicates *p* < 0.01, and *** indicates *p* < 0.001.

In this study, 11 GDH mutants were constructed by site-directed mutagenesis. The pET-28a(+) plasmid, wild-type GDH, and single-point mutations (pET-GDH, pET-P45A, pET-N46E, pET-F155Y, pET-E170R, pET-V227A, pET-W230F, and pET-Q252L) were transformed individually into *E. coli* Bl-21 and expressed at 22°C and 200 rpm for 12 h with the addition of 0.5 mmol/L IPTG. The result of SDS-PAGE indicated that the GDH mutants were solubly expressed in *E. coli* BL-21. Subsequently, a crude enzyme was purified by Ni^2+^ affinity chromatography, and the pure protein with an obvious size of less than 35 kDa (predicted molecular mass of 28.1 kDa), which is consistent with previous research (Figure 2A; Xuejiao, 2011). We detected the activity of single variants by analyzing the generation of NADPH at 340 nm. Comparing the results for the single mutants of all seven of the selected residues, we found that the catalytic activity of P45A, F155Y, and Q252L increased dramatically compared to the wild type, especially P45A whose enzyme activity was improved by roughly 29-fold and reached up to 1829 U/mg (Figure 2B). Although N46E, E170R, V227A, and W230F could lead to at least a 50% increase in thermal stability, the enzyme activity of four single mutants did not change (Vazquez-Figueroa et al., 2007). To further improve the catalytic ability of GDH, P45A, F155Y, and Q252L were combined and further analyzed. However, the effect of mutation P45A dominates the effect of the other individual substitutions. The following order of increasing activity was deduced: P45A/F155Y/Q252L < P45A/F155Y < P45A/Q252L < P45A. We observed that combining the single mutations did not increase enzyme activity which resulted in a plateau of activity for P45A. In this study, the initial rate of NADPH generation was detected with different NADP^+^ concentrations to analyze the kinetic parameters of GDH mutants. We found that the *K*_m_ value of mutant P45A was remarkably less than that of other mutants, whereas the *K*_cat_/*K*_m_ value was at least seven-fold higher (Table 2). Notably, the *K*_cat_/*K*_m_ value of P45A was much higher than that of glucose dehydrogenase from *Bacillus* sp. G3 and *B. megaterium* (Xuejiao, 2011; Qian et al., 2020). We speculated that it may be partially due to the more hydrogen bonds formed by P45A with NADP^+^. There are seven hydrogen bonds formed in the P45A-NADP^+^ complex, including four hydrogen bonds by Asp65, Tyr39, Ans41 and Ser17 with H-50, O-13, O-15 and O-20 of NADP^+^ (2.3 Å, 1.9 Å, 1.9 Å and 2.3 Å), and three hydrogen bonds by Ser40 with O-15 and O-16 of NADP^+^ (2.5 Å, 2.1 Å and 2.3 Å; Supplementary Table 9). According to the results shown above, we surmise that residue Ser40 plays a crucial role in the catalytic activity and NADP^+^ binding ability of GDH as shown in Figures 1A–D (Rassaei et al., 2012).

**Table 2:** Kinetic parameters of GDH mutants.

	*K*_m_ (μM)	*V*_max_ (μM·min^−1^)	*K*_cat_ (s^−1^)	*K*_cat_/*K*_m_ (mM^−1^·s^−1^)
GDH	110.58	19.72	39.44	356.66
P45A	6.09	909.09	1,818.18	298,551.7
F155Y	15.13	322.58	645.16	42641.11
Q252L	74	1,111.11	2,222.22	30,030
P45A-F155Y	161.25	1,250	2,500	15,505.88
P45A-Q252L	104.29	1,428.57	2,857.14	27,396.11
F155Y-Q252L	91.83	1,666.67	3,333.34	36,299.03
P45A-F155Y-Q252L	106.32	1,124.54	2,249.08	21,153.88

### Hydrophobicity analysis of GDH mutants

**Figure 3:**
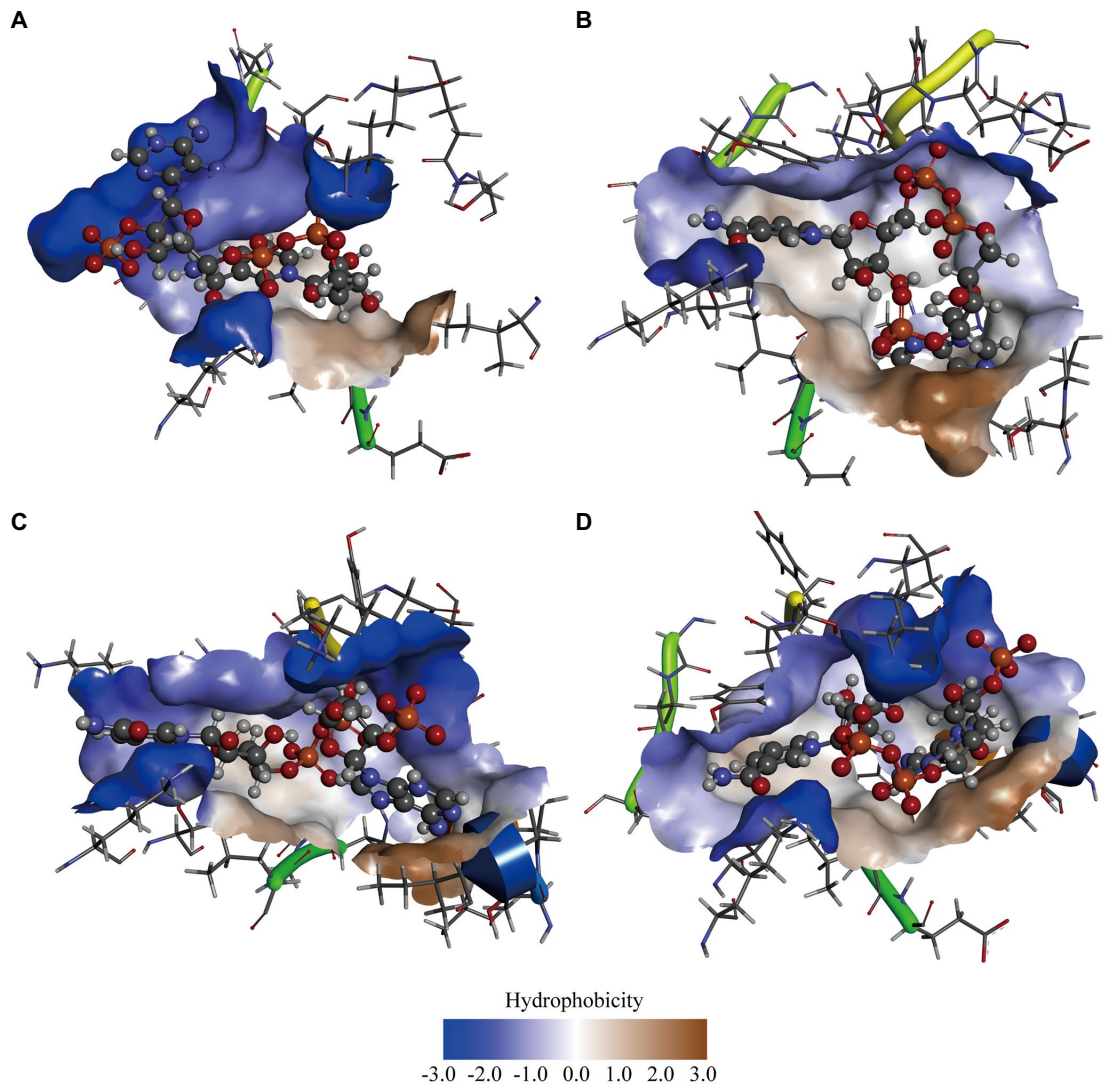
Computer-assisted hydrophobicity analysis of GDH mutants. **(A)** GDH-NADP^+^ complex; **(B)** P45A-NADP^+^ complex; **(C)** N46E-NADP^+^ complex; **(D)** F155Y-NADP^+^ complex. The shade of color indicates the strength of hydrophobicity.

In previous studies, it was suggested that the thermal stability of mutants Q252L and E170K was improved by strengthening the hydrophobic interactions at the dimer-dimer interface (Baik et al., 2005). Rafael et al. improved the activity of peroxidase by increasing the superficial hydrophobicity of the enzyme and found that its activity was correlated with enzyme hydrophobicity (Vazquez-Duhalt et al., 1992). Therefore, we speculated that GDH activity may also be related to protein hydrophobicity. To test our hypothesis, the three most significant mutations (P45A, F155Y, and N46E) were chosen in this study. First, we predicted the hydrophobicity of the protein-ligand complexes and found that compared with wild-type GDH, the pocket hydrophobicity of the three mutants changed significantly (Figure 3). It was notable that the pocket hydrophobicity of the P45A-NADP^+^ complex was enhanced (Figures 3A,B), which is consistent with our previous speculation.

**Figure 4:**
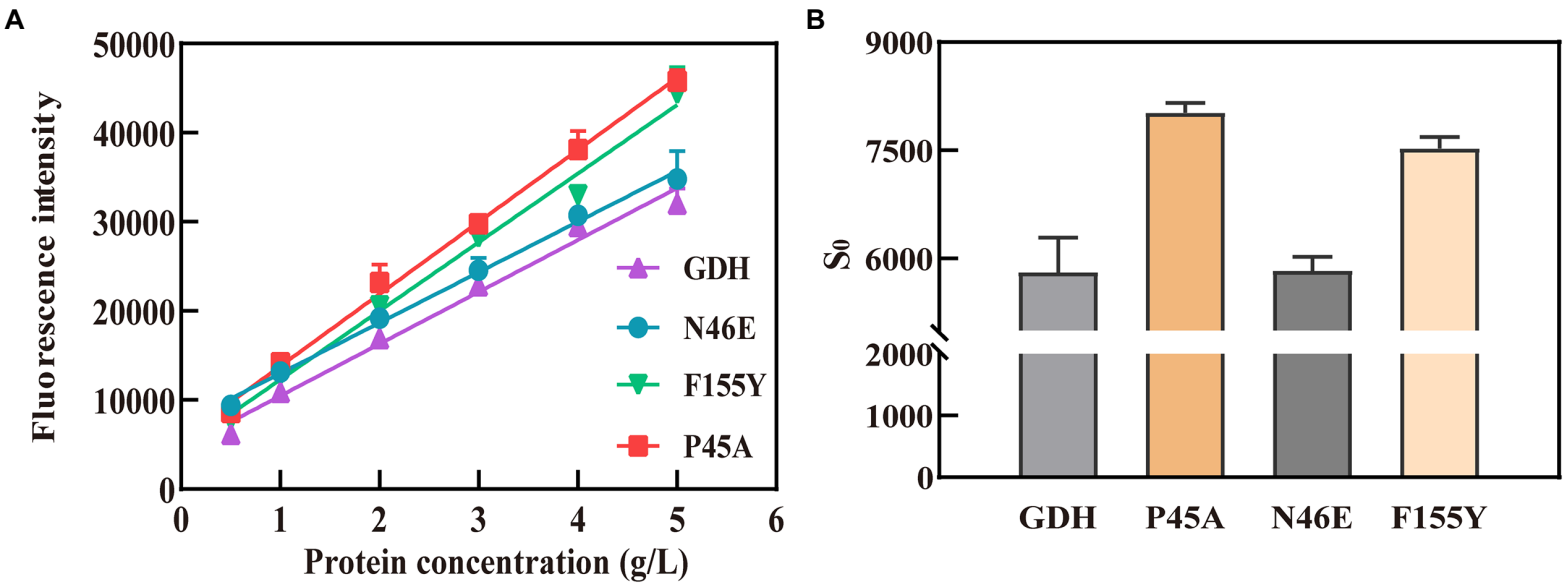
Hydrophobicity analysis of GDH mutants by ANS fluorescent probe. **(A)** Fitting curves for GDH mutants. The fluorescence intensity (λ_ex_ 390 nm and λ_em_ 504 nm) of the GDH variants was analyzed and fitted linearly. **(B)** Hydrophobicity index (S_0_) of GDH mutants. The curve slope was used as an index of protein hydrophobicity (S0). The results are expressed as the mean ± SD (*n* = 3).

Subsequently, an ANS fluorescent probe was used to detect the hydrophobicity of protein, which can significantly increase the fluorescence quantum yield after binding with hydrophobic regions of proteins (Stryer, 1965). Above all, we formulated 0.6 g/L pure GDH using Tris–HCl buffer (pH 7.4) and examined its fluorescence spectra to confirm its excitation and emission wavelengths. The optimal wavelength was 390 nm for excitation and 504 nm for emission, according to wavelength scanning results (Supplementary Figure 9), and were used for the subsequent studies. The concentration of pure GDH variants ranged from 0.5 to 5 g/L after dilution with 20 mM Tris–HCl buffer (pH 7.4, containing 500 mM NaCl). The fluorescence intensity (*λ*_ex_ 390 nm and *λ*_em_ 504 nm) of the GDH variants was analyzed and fitted linearly (Figure 4A). The curve slope was used as an index of protein hydrophobicity (S_0_). The hydrophobicity index (S_0_) of GDH, P45A, F155Y, and N46E was 5,674, 8,091, 7,708, and 5,830, respectively (Figure 4B; Supplementary Table 10). Meanwhile, SDS binding method and curcumin were also used to detect the hydrophobicity of protein (Kato et al., 2002; Sneharani, 2016). The results further demonstrated that the hydrophobicity of P45A is stronger than other GDH mutants (Supplementary Figure 11), which is consistent with our previous findings. This suggests that the enzymatic activity of GDH is positively correlated with its pocket hydrophobicity (Figures 2B, 4B; Supplementary Figure 10). Despite this report on the relation between the two parameters in GDH, the mechanism remains unclear.

### Characteristics of GDH and P45A

**Figure 5:**
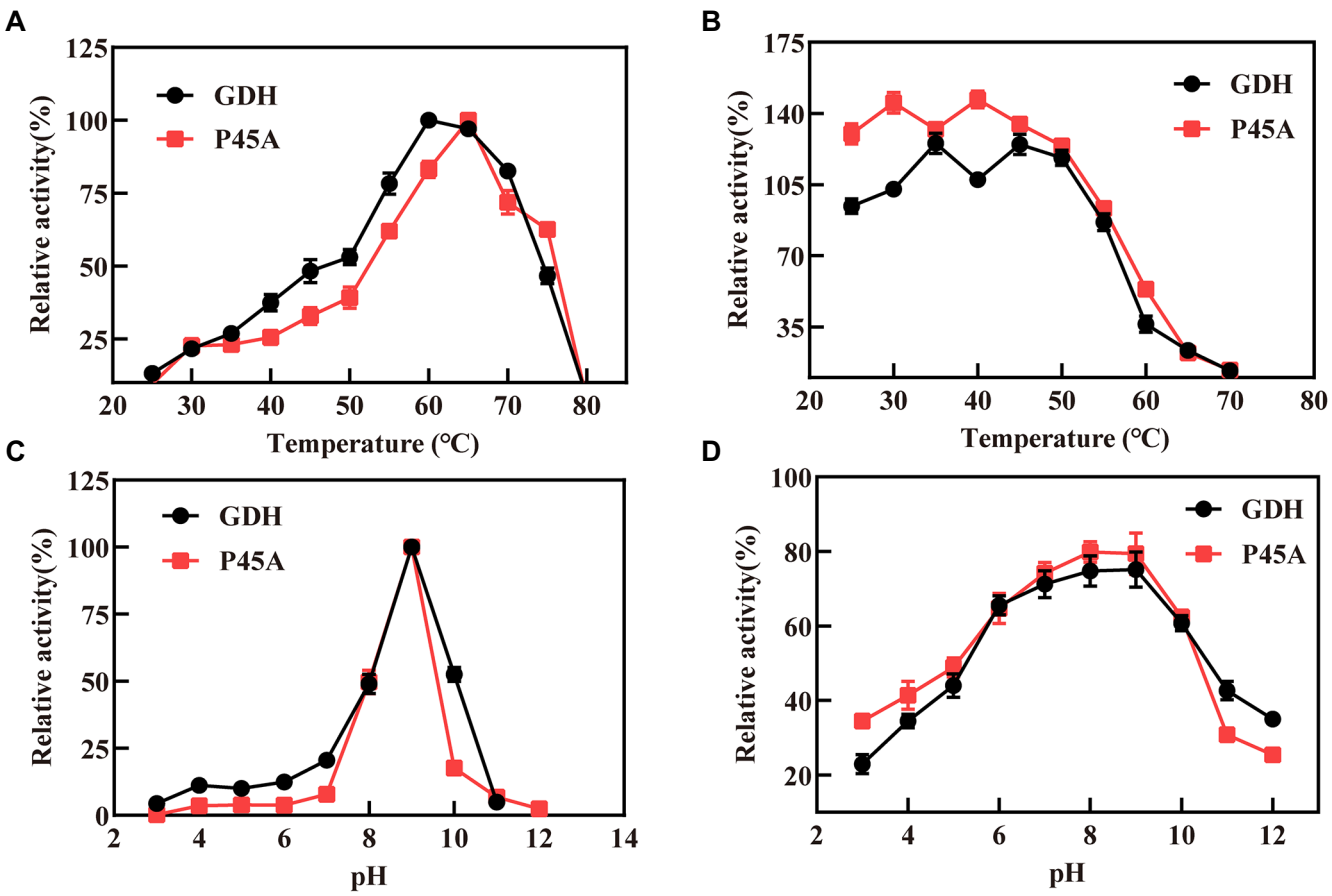
Characteristics of GDH and P45A. **(A)** Effect of different temperature values on the activity of GDH and P45A; **(B)** The residual activity of GDH and P45A after incubated at various temperatures for 20 min; **(C)** Effect of different pH values on the activity of GDH and P45A; **(D)** The residual activity of GDH and P45A after incubated at various pH values for 20 min. The results are expressed as the mean ± SD (*n* = 3).

In this study, we analyzed the characteristics of P45A because it had better catalytic ability than other mutants. Figure 5 shows the relative activity of GDH and P45A at different pH and temperature. According to the data presented in Figure 5A, the replacement of the proline residue by alanine increased the temperature optimum from 60°C to 65°C, which is much higher than that of *B. amyloliquefaciens* SB5 with NADP^+^ as the substrate (Pongtharangkul et al., 2015). This indicated that the P45A mutant has a greater potential in catalytic temperature than most *Bacillus* GluDHs, which exhibited almost no activity at 60°C (Yamamoto et al., 1990; Nagao et al., 1992; Hyun et al., 2016). The thermal stability did not change significantly through site-directed mutagenesis. It was discovered that GDH and P45A were stable at 55°C, and the remaining activity reached 80% after 20 min of treatment, as shown in Figure 5B. However, the residual activity decreased to 30% after 20 min treatment at 60°C, which suggests that the thermal stability of glucose dehydrogenase needs further research.

The optimum pH for purified GDH and P45A was determined by measuring the activity at 60°C. The pH ranged from 3 to 12. We found that the optimal pH of the purified GDH and P45A was observed at pH 9 (Figure 5C), which was lower than the pH 10.0 generally reported for alkali-resistant GluDH from *Lysinibacillus sphaericus* G10 (Ding et al., 2011), and *B. amyloliquefaciens* SB5 (Pongtharangkul et al., 2015). In this study, pH stability was evaluated by keeping the purified enzyme at a specified pH, which is presented as the percentage of remaining specific activity after 20 min (Figure 5D). The residual enzyme activity was over 80% at pH 7.0–9.0, which is better than other GDHs from *Bacillus* species (Mitamura et al., 1989; Yamamoto et al., 1990; Hilt et al., 1991; Nagao et al., 1992). However, the enzyme activity decreased noticeably at pH ≥ 10.0. This is because the GDH tetramer dissociates into inactive monomers at higher pH as a result of the repulsion of acidic amino acids located at the subunit-subunit interface (Nagao et al., 1989; Baik et al., 2003).

### Effect of organic solvents on the catalytic activity and stability of GDH and P45A

**Figure 6:**
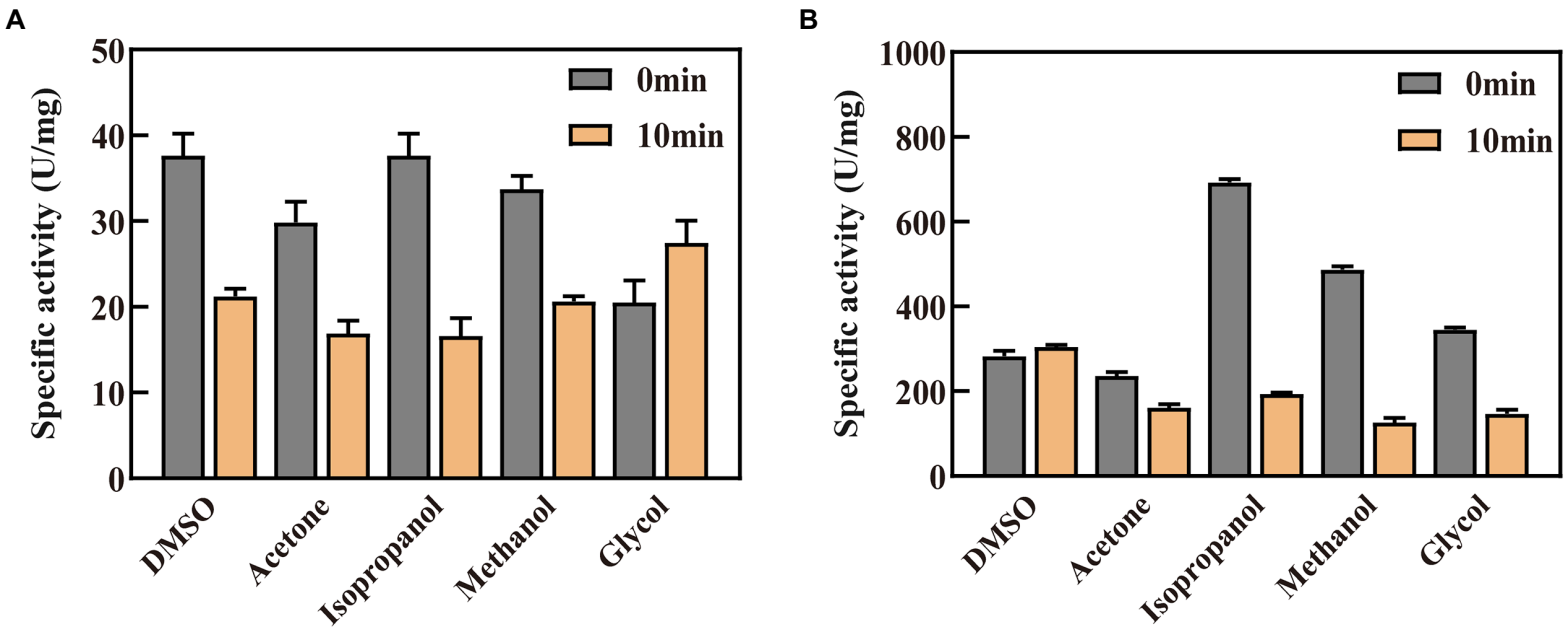
Effect of organic solvents on the catalytic activity and stability of GDH and P45A. **(A)** Effect of organic solvents on GDH. **(B)** Effect of organic solvents on P45A. A final concentration of 10% (V/V) organic solvent was added into the enzyme solution. The residual enzyme activity was detected when the enzyme was kept in the solution for 0 or 10 min. The results are expressed as the mean ± SD (*n* = 3).

The organic solvent tolerance of the enzyme is an important indicator for building *in vitro* synthetic biology platforms of chiral chemicals and constructing organic phase enzyme sensors in non-aqueous systems (Liszka et al., 2012). In this study, the enzyme activity was detected in the buffer system after adding five specific organic solvents (Figure 6). It was discovered that GDH and P45A displayed the best activity in 10% isopropanol solvent, and reached 36.61 and 691.6 U/mg, respectively. According to the results obtained, P45A still showed better catalytic ability than wild-type GDH in organic solvents. However, the enzyme activity of P45A decreased significantly after 10 min in a solution of isopropanol, methanol, and glycol (Figure 6B), which was greater than that of GDH (Figure 6A). It may be that the hydrated layer and salt bridges of P45A were more easily destroyed than that of GDH, these are the major forces maintaining the structure of the protein (Xu et al., 2013). On the other hand, the active pocket of P45A was more easily attacked by polar organic solvents as its higher hydrophobicity led to a decrease in the substrate binding ability (Dumorne et al., 2017).

Subsequently, kinetic constants and the half-life in organic solvents (*t*_1/2_) of the two enzymes were detected and are shown in Table 3. We found that the maximum reaction rate (*V*_max_), fast reaction rate (*K*_cat_), and *K*_cat_/*K*_m_ for P45A were much higher than that of wild-type GDH in all five organic solvents. In addition, its half-life in organic solvents (*t*_1/2_) was longer than that of wild-type GDH. This indicated that the stability of P45A in organic solvents was also enhanced, which may be because the higher hydrophobicity of P45A was more conducive to forming a thermodynamic driving force (Klibanov, 1997; Alsafadi and Paradisi, 2013). However, higher *K*_m_ values toward NADP^+^ (isopropanol-32.82 μM, acetone-26.58 μM, methanol-238.5 μM, and DMSO-35.21 μM) were observed for P45A in comparison with GDH (18.47, 12.62, 15.43, and 16.42 μM, respectively), suggesting that its substrate affinity was affected by organic solvents. In the future, we will focus on improving the substrate affinity of GDH in organic solvents.

**Table 3:** Kinetic parameters of GDH and P45A in organic solvents.

Organic solvent (V/V, 10%)	Enzyme	*K*_m_ (μM)	*V*_max_ (μM·min^−1^)	*K*_cat_ (s^−1^)	*K*_cat_/*K*_m_ (mM^−1^·s^−1^)	*t*_1/2_ (min)
Isopropanol	GDH	18.47	43.67	87.34	4,728.75	8.92
	P45A	32.82	588.24	1,176.48	35,846.44	8.24
Acetone	GDH	12.62	45.88	91.76	7,271	12.74
	P45A	26.58	833.33	1,666.66	62,703.54	22.73
Methanol	GDH	15.43	40.16	80.32	5,205.44	14.15
	P45A	238.5	2,500	5,000	20,964.36	14.56
Glycol	GDH	17.73	45.46	90.92	5,128.03	19.47
	P45A	15.38	625	1,250	81,274.38	23.42
DMSO	GDH	16.42	60.61	121.22	7,382.46	13.17
	P45A	35.21	714.29	1,428.58	40,573.13	19.8

## Conclusion

In conclusion, we successfully obtained an excellent mutant P45A by site-directed mutagenesis and found that the catalytic activity of GDH was positively correlated with the hydrophobicity of its active pocket. The synthesis of NADPH by GDH is restricted by many factors, including but not limited to hydrogen bonds in the protein-ligand complex and the thermal stability of the biocatalyst. In this study, the enzymatic activity of mutant P45A was significantly improved by 29-fold, to 1829.03 U/mg. This is because the substrate NADP^+^ affinity and pocket hydrophobicity of mutant P45A were significantly enhanced. This is the first study that reports a positive correlation between pocket hydrophobicity and the catalytic ability of GDH. Meanwhile, P45A can still exert good catalytic ability in 10% organic solvents. However, its substrate affinity was significantly affected by organic solvents. Therefore, we will focus on improving the substrate affinity of GDH in organic solvents in the future. Our research provides a good theoretical foundation for further improving the catalytic efficiency of GDH.

## Data availability statement

The original contributions presented in the study are included in the article/Supplementary material, further inquiries can be directed to the corresponding authors.

## Author contributions

LH, BQ, ZJ, XY, YS, XW, SY, and LY were involved in the conception and design of the study. BQ, ZJ, and XW performed experimental work and collected data. LH and BQ did data curation and interpretation. LH, XY, and BQ wrote the original draft of the manuscript. LY, SY, XY, and YS contributed in terms of article structuring and editing. All authors contributed to the article and approved the submitted version.

## Funding

This research was financed by the Natural Science Foundation of Henan (202300410055), Postgraduate Cultivating Innovation and Quality Improvement Action Plan of Henan University (SYLYC2022103), and Henan key research and development and promotion special (science and technology, 192102110179).

## Conflict of interest

The authors declare that the research was conducted in the absence of any commercial or financial relationships that could be construed as a potential conflict of interest.

## Publisher’s note

All claims expressed in this article are solely those of the authors and do not necessarily represent those of their affiliated organizations, or those of the publisher, the editors and the reviewers. Any product that may be evaluated in this article, or claim that may be made by its manufacturer, is not guaranteed or endorsed by the publisher.
