# Supplementary material for: Directed evolution engineering to improve activity of glucose dehydrogenase by increasing pocket hydrophobicity
Source: Front Microbiol. 2022 Nov 9;13:1044226. doi: 10.3389/fmicb.2022.1044226 (PMC9681798; doi:10.3389/fmicb.2022.1044226)
Supplement: Supplementary file 1 [file Data_Sheet_1.PDF]

Supplementary Material

Supplementary Tables ••2-19

Supplementary Table 1: GDH flexible docking parameters.

Supplementary Table 2: P45A flexible docking parameters.

Supplementary Table 3: N46E flexible docking parameters.

Supplementary Table 4: F155Y flexible docking parameters.

Supplementary Table 5: E170R flexible docking parameters.

Supplementary Table 6: V227A flexible docking parameters.

Supplementary Table 7: W230F flexible docking parameters.

Supplementary Table 8: Q252L flexible docking parameters.

Supplementary Table 9: Interaction of GDH mutants with $NADP^+$.

Supplementary Table 10: Linear fitting of fluorescence intensities by GDH mutants.

Supplementary Figures ••18-25

Supplementary Figure 1: The structure prediction of GDH by AlphaFold2.

Supplementary Figure 2: The structure prediction of P45A by AlphaFold2.

Supplementary Figure 3: The structure prediction of N46E by AlphaFold2.

Supplementary Figure 4: The structure prediction of F155Y by AlphaFold2.

Supplementary Figure 5: The structure prediction of E170R by AlphaFold2.

Supplementary Figure 6: The structure prediction of V227A by AlphaFold2.

Supplementary Figure 7: The structure prediction of W230F by AlphaFold2.

Supplementary Figure 8: The structure prediction of Q252L by AlphaFold2.

Supplementary Figure 9: Spectra of the GDH fluorescence intensity.

Supplementary Figure 10: Detection of GDH and its hydrophobicity using SDS binding and curcumin.

Supplementary Tables

Supplementary Table 1 GDH flexible docking parameters

Index	(-)CDDOCKER Energy	(-)CDDOCKER interaction Energy	Chiflex energy	Protein conformation	Ionization pH	Libdock score
5	70.51	84.42	-8.92	12	7.5	116.01
9	62.35	78.95	-4.80	20	7.5	103.06
12	59.46	80.39	-8.31	16	7.5	99.52
14	59.25	89.17	-8.34	15	7.5	95.98
26	29.16	53.10	-9.30	10	7.5	94.76
8	62.70	99.37	-8.34	15	7.5	92.86
22	47.66	78.38	-9.35	9	7.5	90.92
11	61.57	85.73	-13.66	1	7.5	88.95
23	44.07	76.44	-9.35	9	7.5	77.66
2	72.31	87.86	-9.05	11	7.5	76.27
24	42.58	60.88	-13.66	1	7.5	74.59
6	70.28	101.90	-13.66	1	7.5	72.84
7	66.82	91.48	-8.12	17	7.5	71.15
3	71.37	74.91	-8.34	15	7.5	67.71
21	49.11	76.74	-8.92	12	7.5	67.57
16	57.85	88.93	-9.79	7	7.5	65.75

27	12.75	38.24	-9.82	6	7.5	65.15
4	70.80	96.85	-9.05	11	7.5	64.66
20	50.69	87.47	-10.47	4	7.5	62.69
17	54.78	81.21	-7.15	19	7.5	62.21
25	32.99	53.92	-1.38	24	7.5	57.30
18	52.85	59.35	-9.48	8	7.5	54.93
10	61.69	85.52	-9.82	6	7.5	50.89
13	59.38	83.97	-8.44	14	7.5	49.74
15	58.19	90.67	-9.82	6	7.5	49.24
19	51.63	66.20	-8.59	13	7.5	30.94

Supplementary Table 2 P45A flexible docking parameters

Index	(-)CDDOCKER Energy	(-)CDDOCKER interaction Energy	Chiflex energy	Protein conformation	Ionization pH	Libdock score	E_{total}	E_{elec}	E_{vdw}
61	41.63	73.52	-8.40	7	7.5	142.38	-15.95	-4.04	-21.45
8	63.38	77.51	-8.40	7	7.5	129.90	-27.00	-9.95	-25.20
38	49.02	71.04	-9.71	3	7.5	128.68	1.47	-6.25	-10.30
10	61.80	81.60	-0.93	23	7.5	127.56	-4.90	-5.58	-12.94
17	56.96	79.13	-8.40	7	7.5	124.18	4.97	-5.94	-9.54

59	42.58	60.21	-6.27	16	7.5	124.00	-17.77	-10.13	-19.67
57	43.51	54.71	-10.13	2	7.5	122.07	-18.02	-6.81	-20.39
28	53.68	67.60	-6.27	16	7.5	121.01	-20.59	-8.68	-21.22
22	56.38	80.36	-12.60	1	7.5	118.28	-12.79	-7.06	-18.35
43	48.22	82.02	-9.71	3	7.5	116.12	-25.24	-9.14	-25.12
26	54.39	83.84	0.52	25	7.5	115.31	-12.30	-6.34	-16.12
9	63.23	87.75	-7.94	8	7.5	115.24	14.30	-6.36	-11.72
3	67.31	81.24	-4.63	19	7.5	114.21	-23.68	-7.39	-25.10
49	45.03	83.09	0.52	25	7.5	113.92	-9.24	-9.79	-19.28
12	60.24	64.23	-10.13	2	7.5	112.15	-19.94	-5.23	
46	47.62	73.55	-8.71	6	7.5	110.77	-6.12	-5.20	-1.00
7	64.15	81.55	-9.39	4	7.5	109.63	-19.55	-6.07	-23.97
48	45.86	68.23	-8.71	6	7.5	109.51	-20.80	-5.21	-25.00
5	64.86	75.75	-0.93	23	7.5	108.83	-6.70	-7.69	-12.73
64	41.23	56.76	-8.71	6	7.5	106.44	-3.53	-4.98	-14.00
56	43.60	61.83	-10.13	2	7.5	106.40	-17.30	-9.29	-1.00
18	56.77	71.40	-9.39	4	7.5	105.38	-22.20	-8.57	-21.70
33	51.29	74.80	-9.39	4	7.5	105.16	27.49	-8.59	1.64
19	56.62	77.34	-6.85	14	7.5	105.07	-16.19	-8.82	-18.79
75	27.16	61.91	-10.13	2	7.5	104.81	-14.08	-7.01	-19.14

15	58.81	74.73	-2.03	21	7.5	104.60	-9.04	-7.81	-14.49
24	56.04	65.69	-12.60	1	7.5	103.10	-16.35	-5.05	-21.00
4	65.33	72.54	-10.13	2	7.5	102.79	-19.07	-7.37	
31	51.89	66.20	-7.94	8	7.5	102.67	-19.71	-7.64	-23.29
6	64.41	89.90	-4.63	19	7.5	102.07	9.99	-5.70	-5.09
16	57.15	62.36	-8.71	6	7.5	102.04	-4.10	-7.63	-14.44
36	50.39	58.06	-8.95	5	7.5	101.29	-22.06	-6.59	
45	48.01	57.48	-7.21	13	7.5	100.63	-2.68	-10.29	-10.30
27	54.00	68.87	-8.95	5	7.5	99.82	-18.89	-8.14	-21.17
21	56.57	76.40	-6.27	16	7.5	98.23	-10.14	-6.00	-15.34
60	41.63	77.57	-2.03	21	7.5	98.06	6.20	-7.23	-13.75
20	56.60	74.42	-6.22	17	7.5	98.03	-11.03	-8.70	-14.75
14	59.46	69.69	-4.63	19	7.5	97.85	-21.34	-7.79	-22.02
42	48.36	62.17	-7.69	10	7.5	97.81	-14.63	-9.04	-16.87
50	44.95	62.48	-0.93	23	7.5	97.64	-23.08	-10.46	-22.00
73	29.74	63.43	-6.22	17	7.5	96.80	0.23	-7.07	-5.60
34	50.96	66.60	-6.53	15	7.5	96.68	-20.01	-8.63	-20.96
23	56.19	80.42	-9.39	4	7.5	96.14	-22.03	-6.52	-23.00
68	39.79	70.79	-6.53	15	7.5	96.07	-18.91	-7.73	-22.81
32	51.77	66.19	-7.94	8	7.5	94.94	-15.54	-6.32	-17.78

71	34.12	59.39	-6.27	16	7.5	94.90	23.97	-5.09	-6.41
53	44.32	57.79	-7.49	11	7.5	94.15	-14.81	-7.53	-19.25
67	40.10	59.97	-6.11	18	7.5	92.96	-14.04	-7.97	-16.88
65	40.76	56.98	-3.77	20	7.5	92.61	-19.78	-7.27	-2.00
47	46.81	75.39	-6.27	16	7.5	91.98	-22.82	-10.06	-2.00
66	40.51	69.92	-6.27	16	7.5	90.57	-10.06	-6.12	-17.27
51	44.74	61.70	-0.93	23	7.5	90.42	-10.51	-7.25	-17.00
41	48.59	60.79	-9.39	4	7.5	89.00	-14.88	-8.88	-16.74
11	60.50	68.70	-6.53	15	7.5	88.94	3.40	-7.31	-16.92
62	41.38	71.01	-2.03	21	7.5	88.94	-19.69	-7.37	-20.87
30	52.27	63.09	-3.77	20	7.5	88.34	-19.01	-6.61	-22.00
72	33.91	68.07	-7.94	8	7.5	88.24	-18.80	-8.35	-20.86
69	38.10	56.05	-7.49	11	7.5	87.13	-13.19	-9.45	-16.21
35	50.80	54.50	-2.03	21	7.5	86.72	2.52	-9.08	-4.41
25	55.64	72.59	-4.63	19	7.5	85.10	6.28	-9.55	-2.50
55	44.08	58.84	-7.88	9	7.5	85.06	14.15	-6.65	-0.47
13	59.47	74.92	-3.77	20	7.5	84.60	-17.32	-12.95	-19.00
54	44.28	65.26	-7.69	10	7.5	80.95	-17.23	-6.75	-19.14
74	28.72	52.08	-7.21	13	7.5	80.62	-23.70	-8.75	-26.43
40	48.81	60.62	0.52	25	7.5	78.35	-19.74	-7.86	-22.51

37	49.46	81.31	-1.66	22	7.5	77.90	-14.68	-4.67	-21.92
63	41.25	57.98	-6.85	14	7.5	77.39	-24.11	-8.48	-24.09
44	48.07	68.86	-2.03	21	7.5	77.33	-15.70	-9.30	-18.35
39	48.94	69.38	-6.27	16	7.5	74.54	-19.26	-7.14	-20.65
29	52.62	81.00	-7.88	9	7.5	74.21	-21.33	-8.61	-21.53
52	44.60	64.46	-1.66	22	7.5	72.76	-21.59	-8.17	-23.34
2	78.79	90.55	-0.45	24	7.5	70.98	-19.23	-8.64	-20.00
70	36.44	52.53	-6.11	18	7.5	70.60	-16.73	-7.54	-18.76
76	23.76	60.83	-0.45	24	7.5	64.76	47.04	-7.52	8.44
58	43.33	65.22	-0.45	24	7.5	59.37	-18.25	-7.87	-20.59

Supplementary Table 3 N46E flexible docking parameters

Index	(-)CDDOCKER Energy	(-)CDDOCKER interaction Energy	Chiflex energy	Protein conformation	Ionization pH	Libdock score	E_{total}	E_{elec}	E_{vdw}
19	55.01	82.65	-13.32	1	7.5	122.18	-11.23	-7.21	-17.67
20	53.91	74.14	-11.34	3	7.5	99.43	-22.37	-5.88	-25.83
3	77.87	82.79	-8.00	19	7.5	98.54	2.28	-7.65	-6.50
8	70.48	93.57	-10.88	5	7.5	98.42	29.29	-9.14	-2.80
7	71.12	96.39	-7.88	20	7.5	96.64	-5.32	-7.21	-12.97
10	67.12	73.82	-11.34	3	7.5	95.94	-17.35	-8.58	-20.79

5	75.39	98.71	-13.32	1	7.5	93.28	-23.78	-6.92	-26.29
21	52.08	78.29	-10.88	5	7.5	91.52	-20.02	-7.01	-22.00
24	46.56	62.65	-8.55	17	7.5	88.40	-21.04	-8.96	-24.27
27	42.52	79.70	-11.34	3	7.5	85.81	-20.80	-9.31	-20.00
15	60.41	94.24	-13.32	1	7.5	83.68	-20.62	-5.50	-23.85
11	61.97	78.76	-9.60	12	7.5	83.38	23.68	-10.63	7.39
23	48.59	69.13	-8.61	15	7.5	79.76	-11.73	-9.47	-14.99
4	76.04	83.93	-9.37	13	7.5	75.60	-9.51	-9.09	-15.80
18	57.22	73.61	-10.88	5	7.5	71.87	-9.98	-6.79	-17.02
25	44.84	70.59	-8.55	17	7.5	70.98	-23.28	-8.32	-25.71
13	61.40	84.02	-11.61	2	7.5	70.04	-23.75	-8.82	-24.10
14	60.91	93.71	-8.36	18	7.5	68.70	-8.14	-10.57	-11.03
6	72.97	82.21	-8.56	16	7.5	68.68	-20.20	-8.84	-22.81
26	44.11	65.66	-9.99	10	7.5	67.30	-20.73	-5.36	-24.54
17	58.15	75.22	-10.01	9	7.5	65.64	-15.36	-9.45	-22.10
12	61.88	94.67	-4.13	23	7.5	63.79	-25.65	-7.18	-28.00
2	84.26	97.49	-8.55	17	7.5	63.03	-21.38	-12.05	-19.81
30	31.56	53.47	-8.36	18	7.5	54.41	-22.82	-9.42	-25.90
9	70.00	79.69	-9.60	12	7.5	53.21	5.88	-6.39	-7.89
29	33.60	65.66	-10.59	6	7.5	42.73	-11.53	-6.68	-18.95

28	38.03	69.38	-9.37	13	7.5	35.54	16.76	-6.20	-13.00
22	48.64	69.23	-9.99	11	7.5	34.87	-15.06	-5.28	-20.45
16	58.84	82.11	-9.37	13	7.5	31.09	-15.03	-7.54	-1.00
19	55.01	82.65	-13.32	1	7.5	122.18	-11.23	-7.21	-17.67
20	53.91	74.14	-11.34	3	7.5	99.43	-22.37	-5.88	-25.83
3	77.87	82.79	-8.00	19	7.5	98.54	2.28	-7.65	-6.50
8	70.48	93.57	-10.88	5	7.5	98.42	29.29	-9.14	-2.80
7	71.12	96.39	-7.88	20	7.5	96.64	-5.32	-7.21	-12.97
10	67.12	73.82	-11.34	3	7.5	95.94	-17.35	-8.58	-20.79
5	75.39	98.71	-13.32	1	7.5	93.28	-23.78	-6.92	-26.29
21	52.08	78.29	-10.88	5	7.5	91.52	-20.02	-7.01	-22.00
24	46.56	62.65	-8.55	17	7.5	88.40	-21.04	-8.96	-24.27
27	42.52	79.70	-11.34	3	7.5	85.81	-20.80	-9.31	-20.00
15	60.41	94.24	-13.32	1	7.5	83.68	-20.62	-5.50	-23.85
11	61.97	78.76	-9.60	12	7.5	83.38	23.68	-10.63	7.39
23	48.59	69.13	-8.61	15	7.5	79.76	-11.73	-9.47	-14.99
4	76.04	83.93	-9.37	13	7.5	75.60	-9.51	-9.09	-15.80
18	57.22	73.61	-10.88	5	7.5	71.87	-9.98	-6.79	-17.02
25	44.84	70.59	-8.55	17	7.5	70.98	-23.28	-8.32	-25.71
13	61.40	84.02	-11.61	2	7.5	70.04	-23.75	-8.82	-24.10

14	60.91	93.71	-8.36	18	7.5	68.70	-8.14	-10.57	-11.03
6	72.97	82.21	-8.56	16	7.5	68.68	-20.20	-8.84	-22.81
26	44.11	65.66	-9.99	10	7.5	67.30	-20.73	-5.36	-24.54
17	58.15	75.22	-10.01	9	7.5	65.64	-15.36	-9.45	-22.10
12	61.88	94.67	-4.13	23	7.5	63.79	-25.65	-7.18	-28.00
2	84.26	97.49	-8.55	17	7.5	63.03	-21.38	-12.05	-19.81
30	31.56	53.47	-8.36	18	7.5	54.41	-22.82	-9.42	-25.90
9	70.00	79.69	-9.60	12	7.5	53.21	5.88	-6.39	-7.89
29	33.60	65.66	-10.59	6	7.5	42.73	-11.53	-6.68	-18.95
28	38.03	69.38	-9.37	13	7.5	35.54	16.76	-6.20	-13.00
22	48.64	69.23	-9.99	11	7.5	34.87	-15.06	-5.28	-20.45
16	58.84	82.11	-9.37	13	7.5	31.09	-15.03	-7.54	-1.00

Supplementary Table 4 F155Y flexible docking parameters

Index	(-)CDDOCKER Energy	(-)CDDOCKER interaction Energy	Chiflex energy	Protein conformation	Ionization pH	Libdock score	E_{total}	E_{elec}	E_{vdw}
8	64.22	81.89	-9.63	9	7.5	129.85	-6.51	-6.04	-13.62
7	65.74	95.97	-11.77	2	7.5	122.87	-14.28	-8.38	-16.68
6	68.94	80.62	-9.15	12	7.5	117.61	-16.18	-7.99	-

16	62.07	69.60	-8.36	14	7.5	114.03	-23.06	-7.86	-25.74
17	59.85	74.38	-8.36	14	7.5	113.80	-21.02	-10.14	-21.25
23	36.64	64.63	-11.77	2	7.5	108.10	-11.38	-6.83	-17.19
3	74.40	86.99	-11.12	3	7.5	105.65	-16.13	-6.85	-
5	71.94	84.67	-7.61	18	7.5	103.97	-16.36	-8.01	-19.78
21	42.67	81.38	-8.36	14	7.5	102.60	-21.31	-7.64	-23.01
15	62.69	75.57	-11.77	2	7.5	101.02	-13.85	-4.89	-
11	63.54	92.53	-9.63	9	7.5	100.14	-22.27	-7.15	-2.00
13	62.94	88.85	-7.61	18	7.5	96.92	-2.17	-5.39	-11.98
20	49.84	80.73	-6.61	19	7.5	96.14	-16.44	-6.30	-20.50
2	82.34	88.17	-6.61	19	7.5	87.87	-18.61	-5.87	-22.00
4	72.82	79.12	-9.67	7	7.5	87.56	-20.32	-7.26	-
14	62.89	87.80	-10.94	4	7.5	86.76	-20.69	-9.99	-21.00
9	64.20	87.77	-1.66	24	7.5	79.88	-15.33	-4.60	-20.73
25	24.01	52.33	-11.77	2	7.5	76.72	18.02	-7.33	-3.00
24	31.90	23.18	-0.77	25	7.5	70.27	-12.98	-6.32	-16.92
12	63.42	87.37	-8.49	13	7.5	66.83	-22.85	-9.63	-23.36
22	40.61	83.63	-9.59	10	7.5	55.43	-27.19	-8.74	-2.00
19	52.41	76.37	-8.49	13	7.5	49.66	-20.79	-6.48	-24.16
18	54.26	86.55	-1.66	24	7.5	21.59	27.20	-8.16	16.15

10	64.15	80.57	-1.66	24	7.5	-162.00	-14.85	-3.91	-21.00

Supplementary Table 5 E170R flexible docking parameters

Index	(-)CDDOCKER Energy	(-)CDDOCKER interaction Energy	Chiflex energy	Protein conformation	Ionization pH	Libdock score	E_{total}	E_{elec}	E_{vdw}
8	62.45	93.12	-11.53	5	7.5	153.40	-21.67	-5.69	-26.06
9	62.39	84.99	-14.84	1	7.5	114.82	-7.45	-7.38	-18.68
4	73.06	100.20	-12.19	3	7.5	107.55	-21.77	-7.73	-2.00
2	80.44	113.51	-8.64	19	7.5	106.69	-14.90	-8.82	-19.50
10	62.28	76.85	-12.53	2	7.5	104.36	-18.73	-7.49	-22.97
12	56.82	87.20	-11.21	6	7.5	101.43	-27.28	-7.81	-30.10
13	53.04	89.36	-11.21	6	7.5	100.49	-27.29	-7.83	-30.10
11	61.59	81.40	-14.84	1	7.5	99.69	1.64	-10.97	-10.23
6	64.84	69.31	-9.05	17	7.5	78.66	-20.01	-7.87	-
5	65.13	84.03	-3.30	23	7.5	71.28	-16.01	-4.16	-21.41
14	52.82	74.82	-9.67	12	7.5	59.24	-27.93	-7.39	-30.19
7	63.96	94.17	-10.40	9	7.5	52.17	-20.76	-7.12	-25.74
3	76.60	98.65	-4.27	22	7.5	-56.87	-20.50	-11.04	-21.29

Supplementary Table 6 V227A flexible docking parameters

Index	(-)CDDOCKER Energy	(-)CDDOCKER interaction Energy	Chiflex energy	Protein conformation	Ionization pH	Libdock score	E_{total}	E_{elec}	E_{vdw}
15	48.15	68.54	-9.33	10	7.5	138.90	-12.31	-8.07	-19.25
4	66.18	95.02	-7.38	17	7.5	124.92	-19.31	-7.55	-22.13
8	60.25	84.78	-9.33	10	7.5	122.58	-3.28	-12.68	-12.30
10	55.12	86.44	-6.93	19	7.5	108.51	-18.87	-9.42	-23.03
13	48.99	79.64	-9.33	10	7.5	102.86	-26.55	-8.18	-
14	48.87	67.86	-7.34	18	7.5	102.49	-19.79	-7.79	-22.00
6	62.82	90.11	-10.07	5	7.5	100.51	-21.29	-7.18	-23.60
16	48.00	86.49	-9.33	10	7.5	100.34	-26.55	-8.31	-2.00
5	63.93	80.13	-9.23	11	7.5	94.91	-13.36	-7.43	-17.18
17	40.18	63.38	-6.93	19	7.5	84.80	-20.84	-5.68	-25.14
11	51.29	61.97	-6.89	20	7.5	78.96	-8.90	-8.97	-13.42
9	58.71	69.94	-6.89	20	7.5	77.46	-11.18	-9.50	-16.27
7	62.05	81.71	-13.95	1	7.5	65.09	-18.32	-5.69	-21.67
12	51.09	76.75	-9.88	6	7.5	57.45	-21.47	-7.79	-23.22
3	68.96	89.15	-8.04	16	7.5	53.72	32.38	-9.12	1.67

2	73.98	97.71	-9.84	7	7.5	46.95	-6.87	-8.39	-11.62

Supplementary Table 7 W230F flexible docking parameters

Index	(-)CDDO CKER Energy	(-)CDDO CKER interaction Energy	Chiflex energy	Protein conformation	Ionization pH	Libdock score	E_{total}	E_{elec}	E_{vdw}
7	67.13	96.38	-10.74	6	7.5	128.26	-21.91	-6.71	-2.00
12	56.60	92.20	-9.90	10	7.5	124.17	0.72	-9.30	-1.00
20	50.95	81.44	-11.91	2	7.5	122.25	-7.07	-8.11	-12.00
6	72.69	105.43	-7.67	19	7.5	103.51	-22.10	-8.23	-25.00
8	66.31	72.26	-10.08	9	7.5	101.88	-20.63	-7.22	-22.50
11	57.02	85.94	-9.90	10	7.5	101.32	3.60	-10.14	-4.00
19	51.13	69.26	-7.74	18	7.5	100.92	-21.31	-8.41	-23.30
2	87.46	103.99	-11.17	4	7.5	100.66	-29.93	-11.45	-27.00
28	41.17	64.18	-7.74	18	7.5	100.01	-22.99	-7.49	-26.00
16	54.00	91.51	-9.90	10	7.5	99.75	5.10	-9.50	-9.30
10	59.14	75.53	-9.88	12	7.5	98.05	-18.24	-7.25	
23	47.99	81.69	-10.36	8	7.5	97.56	-16.93	-5.80	-2.00
13	56.50	65.96	-11.91	2	7.5	97.29	-3.75	-5.39	
26	46.29	70.51	-11.91	2	7.5	96.68	-21.40	-8.06	-2.00

15	55.37	89.63	-9.20	16	7.5	95.74	-23.48	-6.57	-26.20
18	52.50	74.39	-7.74	18	7.5	93.58	-17.75	-9.50	-19.10
3	79.22	91.81	-9.13	17	7.5	91.94	-9.83	-7.76	
17	53.34	78.01	-7.01	20	7.5	89.19	-9.15	-10.25	-13.00
22	48.78	78.12	-9.90	11	7.5	88.72	-19.86	-7.83	-2.00
14	56.19	84.12	-6.43	21	7.5	85.91	-23.80	-6.81	
25	46.69	76.71	-10.36	8	7.5	85.42	-15.86	-8.00	-20.31
5	72.74	103.77	-11.09	5	7.5	80.06	-22.06	-4.96	
4	73.32	95.47	-9.41	13	7.5	76.78	-23.55	-10.76	-24.16
30	25.95	51.66	-4.04	23	7.5	73.64	-8.49	-4.29	
24	47.90	80.83	-4.04	23	7.5	66.02	-20.34	-6.72	-22.00
21	49.98	70.48	-4.04	23	7.5	56.15	-11.67	-5.09	
29	30.72	54.83	-2.58	25	7.5	55.76	26.59	-9.26	9.24
27	46.12	61.82	-2.99	24	7.5	49.17	-13.57	-6.41	-16.00
9	63.11	83.53	-5.14	22	7.5	38.21	-16.83	-5.33	-21.20

Supplementary Table 8 Q252L flexible docking parameters

Index	(-)CDDOCKER Energy	(-)CDDOCKER interaction Energy	Chiflex energy	Protein conformation	Ionization pH	Libdock score	E_{total}	E_{elec}	E_{vdw}
25	55.60	81.73	-9.33	9	7.5	121.72	-11.96	-9.95	-18.10

21	58.18	98.81	-7.48	17	7.5	115.89	-19.79	-9.00	-24.07
7	71.22	76.04	-7.48	17	7.5	113.93	9.88	-9.70	-10.33
14	62.66	85.50	-9.33	9	7.5	106.46	-15.62	-5.01	-20.33
23	56.14	76.24	-10.47	4	7.5	106.03	-20.52	-7.43	-23.20
4	77.50	88.10	-8.36	14	7.5	103.38	-21.78	-7.54	-24.54
16	60.73	74.65	-10.13	5	7.5	96.36	40.80	-7.02	10.07
11	66.82	91.52	-9.26	11	7.5	93.13	-14.98	-8.92	-19.24
30	47.57	64.72	-9.33	9	7.5	92.98	-10.55	-8.16	-15.80
3	81.49	98.87	-8.12	16	7.5	92.16	-17.84	-4.82	-24.53
28	49.92	74.93	-11.23	2	7.5	88.08	-4.31	-7.52	-11.00
9	68.71	90.81	-9.33	9	7.5	88.03	-25.85	-12.34	-22.30
24	55.98	79.01	-8.30	15	7.5	83.29	-11.80	-9.38	-21.58
8	69.79	88.51	-5.83	19	7.5	81.70	-10.85	-8.08	-17.00
13	65.12	69.89	-14.13	1	7.5	81.68	-18.47	-8.72	-20.83
19	58.82	88.38	-9.67	6	7.5	80.97	7.12	-5.73	-9.88
27	53.16	77.85	-14.13	1	7.5	78.74	-24.15	-8.08	-24.87
15	61.55	91.90	-8.30	15	7.5	73.71	-15.61	-7.17	-19.35
26	55.51	78.81	-10.83	3	7.5	72.66	-21.73	-9.88	-22.56
20	58.55	72.55	-11.23	2	7.5	68.64	-18.08	-8.05	-1.00
6	71.79	87.71	-8.36	14	7.5	67.40	-15.83	-6.58	-20.11

10	67.10	87.42	-9.27	10	7.5	66.83	-18.58	-6.39	-23.14
5	73.55	94.31	-2.59	22	7.5	65.38	12.56	-7.13	-8.60
17	59.85	78.36	-10.13	5	7.5	65.00	39.48	-7.16	0.89
22	56.17	83.32	-10.47	4	7.5	63.85	-21.96	-7.36	
31	42.52	79.93	-8.30	15	7.5	61.06	-23.13	-8.14	-23.98
12	66.34	90.03	-10.47	4	7.5	58.50	-13.88	-8.44	-17.29
2	85.68	88.86	-10.83	3	7.5	51.74	-17.33	-7.38	-20.42
18	59.28	66.96	-9.67	7	7.5	51.30	-19.10	-8.96	-22.04
33	35.06	58.79	-8.52	12	7.5	48.77	10.49	-9.54	-0.86
34	30.27	52.01	-9.51	8	7.5	47.99	3.83	-4.64	-7.23
29	48.20	72.58	-2.20	23	7.5	23.15	-20.72	-6.25	-24.52
32	38.92	56.36	-1.49	25	7.5	-0.84	0.16	-5.23	-10.64

Supplementary Table 9 Interaction of GDH mutants with $NADP^+$

	Number of hydrogen bonds	Combine the location	Distance （Å）		Number of hydrogen bonds	Combine the location	Distance （Å）
		Asp65-H50	2.2			Asp65-H50	2.3
		Gly94-O45	2.6			Lys111-O16	2.2
GDH	5	Lys42-O15	1.7	F155Y	5	Lys42-O15	1.9
		Lys111-O20	1.8			Gln43-O39	2.1
		Lys68-O39	2.4			Gln43-O40	2.5
		Asp65-H50	2.3			Ile195-O16	2.6
		Tyr39-O13	1.9			Leu19-O19	2.2
		Ser40-O15	2.5			Gly20-O19	1.8
P45A	7	Ans41-O15	1.9			Ans92-O19	2.0
		Ser40-O16	2.1;2.3	W230F	8	Ser17-H48	2.7
		Ser17-O20	2.3			Ser17-O41	2.1
		Lys111-O3	2.5			Tyr39-O40	2.0
N46E	3	Tyr39-O20	1.8			Gly94-O24	1.9
		Ile195-N33	2.9				
		Gly94-O39	2.8			Lys68-O47	2.3
		Gln43-O43	2.1			Tyr39-O15	2.3
		Tyr39-O39	2.5			Tyr39-O17	2.2
V227A	7	Gln43-O15	1.9	Q252L	6	Ser40-O40	2.3
		Tyr39-O16	2.0			Lys42-O39	1.9
		Ser40-O17	2.9			Ser17-H65	2.1
		Ser40-O19	2.1				

		Gly94-O3	2.0
		Gly94-O20	2.3
E170R	5	Gln43-O47	2.6
		Tyr39-O15	2.0
		Tyr39-O40	1.5

Supplementary Table 10 Linear fitting of fluorescence intensities by GDH mutants

Mutants	Linear fit equation	R^2
GDH	y = 5673.9x + 7311.2	0.995
P45A	y = 8091x + 5699.9	0.997
N46E	y = 5830.8x + 4626.5	0.987
F155Y	y = 7708.6x + 4574.8	0.991

Supplementary Figures

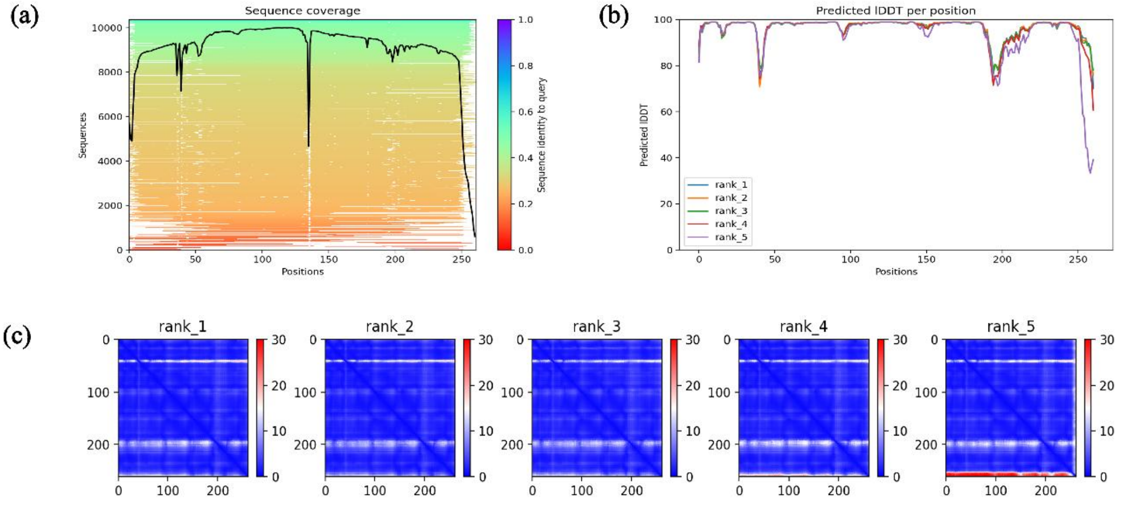

Supplementary Figure 1 The structure prediction of GDH by AlphaFold2. To help researchers judge the predicted structure quality, we visualize multiple sequence alignment (MSA) depth and diversity and show the AlphaFold2 confidence measures (pLDDT and PAE) Error (PAE).

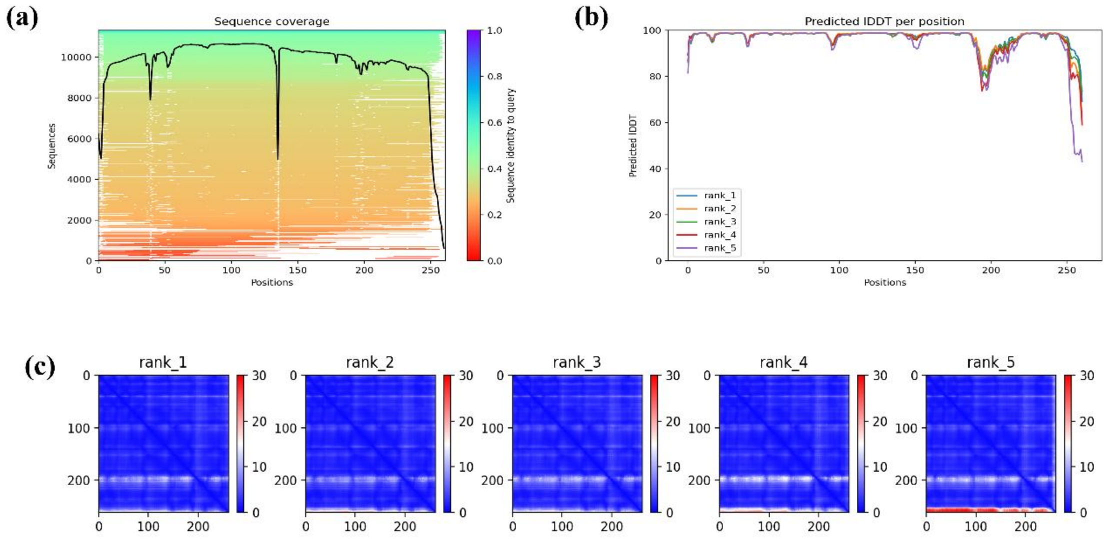

Supplementary Figure 2 The structure prediction of P45A by AlphaFold2. To help researchers judge the predicted structure quality, we visualize multiple sequence alignment (MSA) depth and diversity and show the AlphaFold2 confidence measures (pLDDT and PAE) Error (PAE).

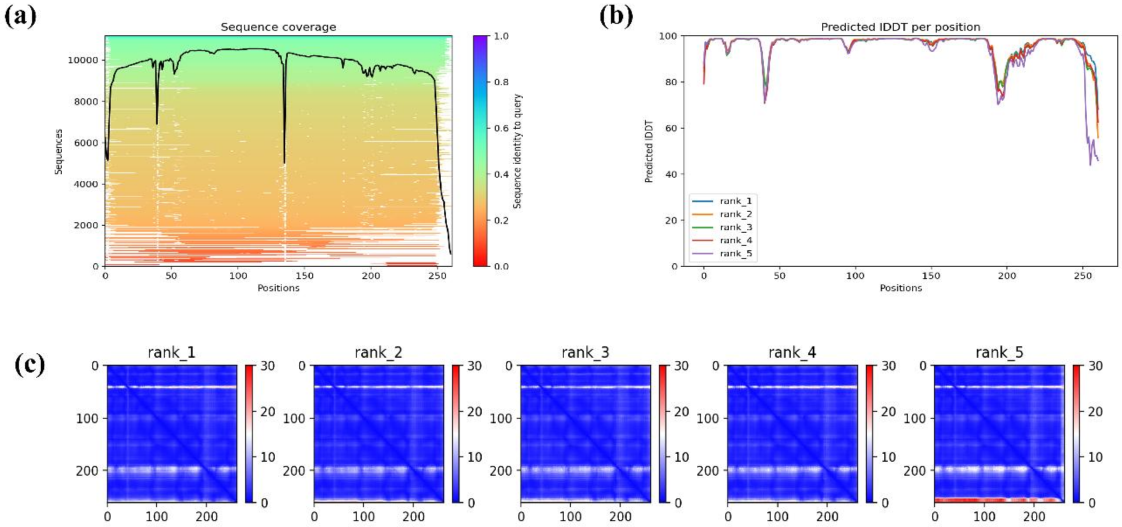

Supplementary Figure 3 The structure prediction of N46E by AlphaFold2. To help researchers judge the predicted structure quality, we visualize multiple sequence alignment (MSA) depth and diversity and show the AlphaFold2 confidence measures (pLDDT and PAE) Error (PAE).

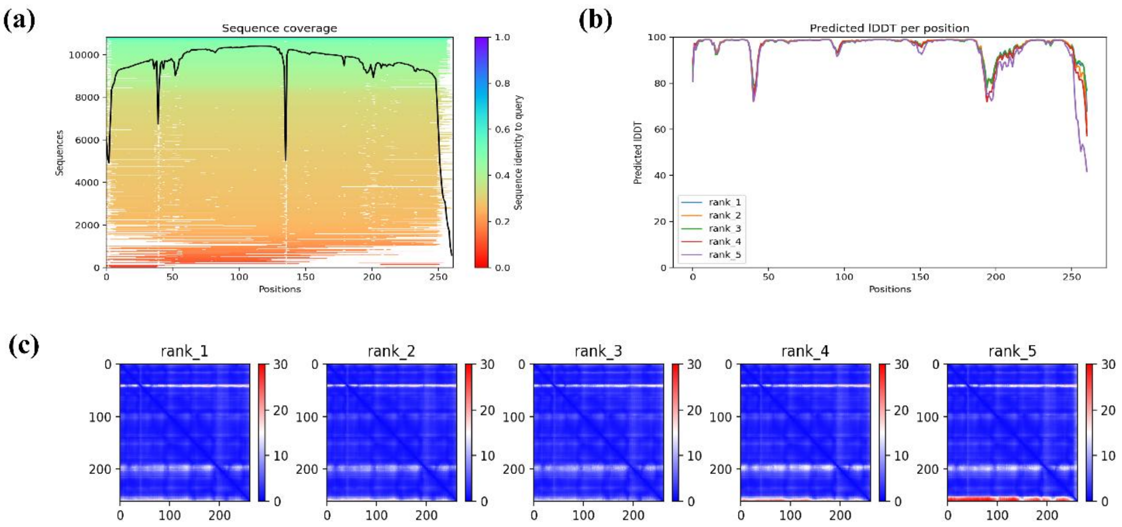

Supplementary Figure 4 The structure prediction of F155Y by AlphaFold2. To help researchers judge the predicted structure quality, we visualize multiple sequence alignment (MSA) depth and diversity and show the AlphaFold2 confidence measures (pLDDT and PAE) Error (PAE).

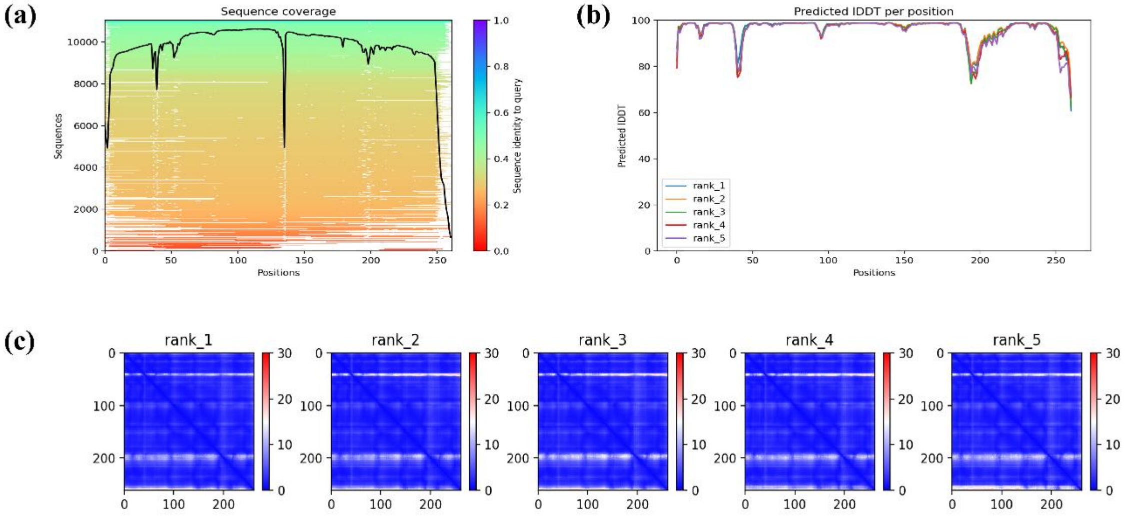

Supplementary Figure 5 The structure prediction of E170R by AlphaFold2. To help researchers judge the predicted structure quality, we visualize multiple sequence alignment (MSA) depth and diversity and show the AlphaFold2 confidence measures (pLDDT and PAE) Error (PAE).

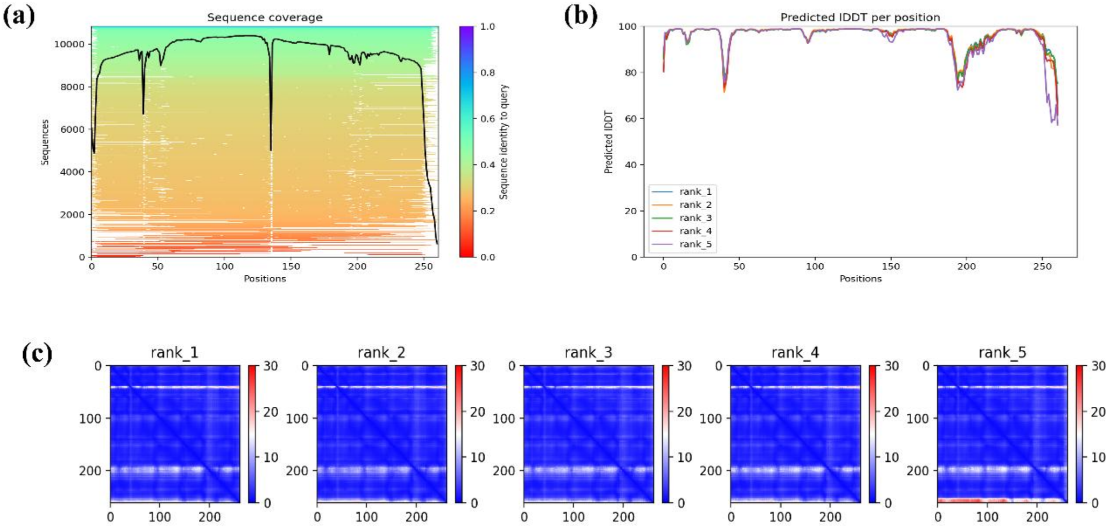

Supplementary Figure 6 The structure prediction of V227A by AlphaFold2. To help researchers judge the predicted structure quality, we visualize multiple sequence alignment (MSA) depth and diversity and show the AlphaFold2 confidence measures (pLDDT and PAE) Error (PAE).

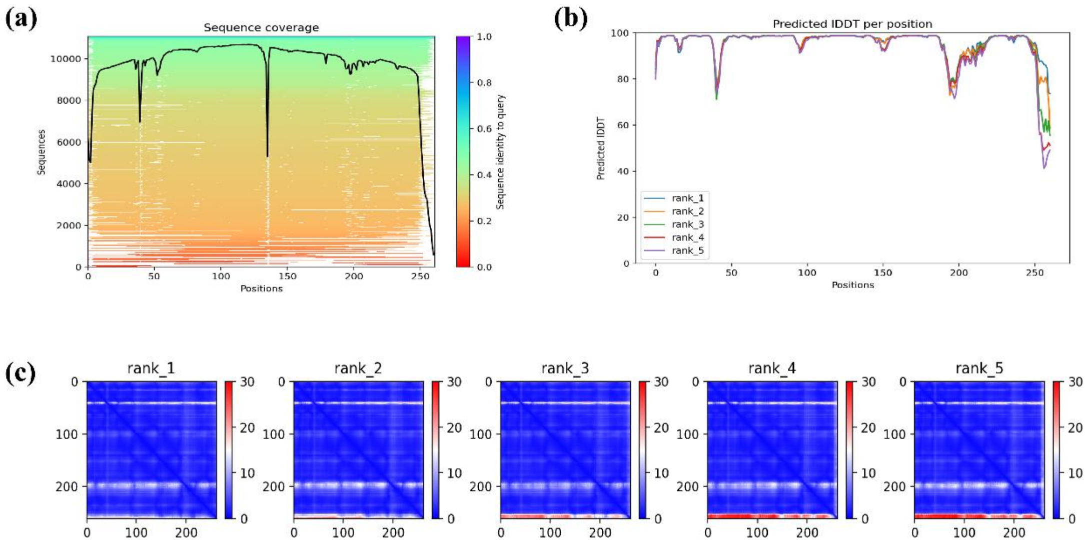

Supplementary Figure 7 The structure prediction of W230F by AlphaFold2. To help researchers judge the predicted structure quality, we visualize multiple sequence alignment (MSA) depth and diversity and show the AlphaFold2 confidence measures (pLDDT and PAE) Error (PAE).

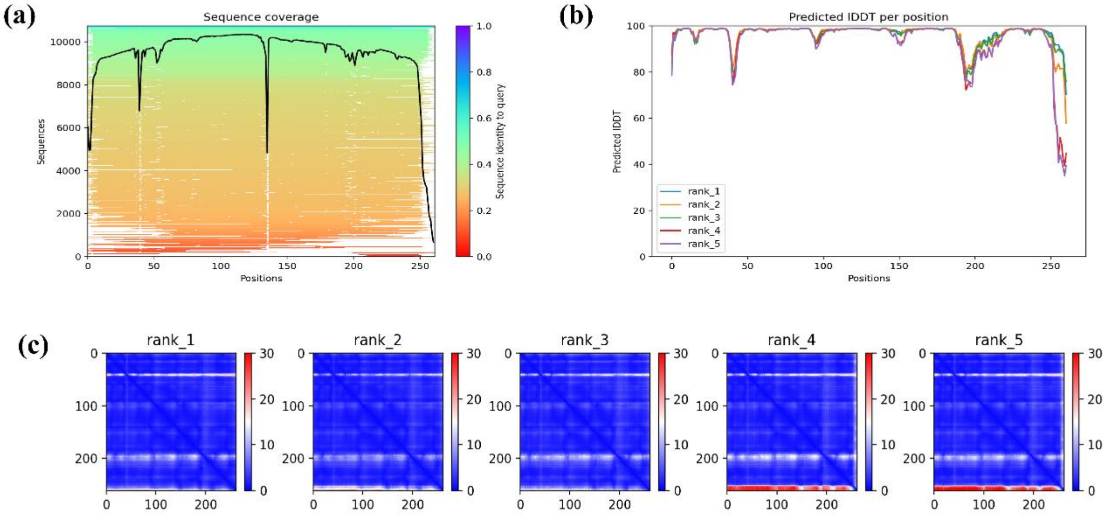

Supplementary Figure 8 The structure prediction of Q252L by AlphaFold2. To help researchers judge the predicted structure quality, we visualize multiple sequence alignment (MSA) depth and diversity and show the AlphaFold2 confidence measures (pLDDT and PAE) Error (PAE).

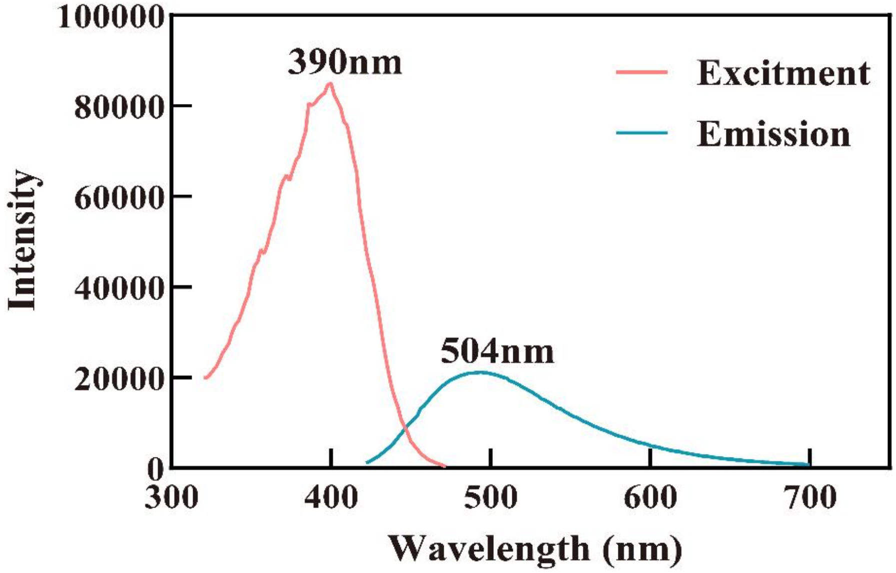

Supplementary Figure 9 Spectra of the GDH fluorescence intensity. The ANS-prepared GDH mixture was used to conduct wavelength scanning with a fluorescence photometer to determine the fluorescence emission spectra. The excitation wavelength was set at 390 nm. The scanning wavelength ranged from 400 to 700 nm. The optimum emission wavelength was analyzed by scanning the emission spectra. The scan range was 300-500 nm, and the emission wavelength was set at 504 nm. To find the ideal excitation wavelength, the excitation spectrum was examined.

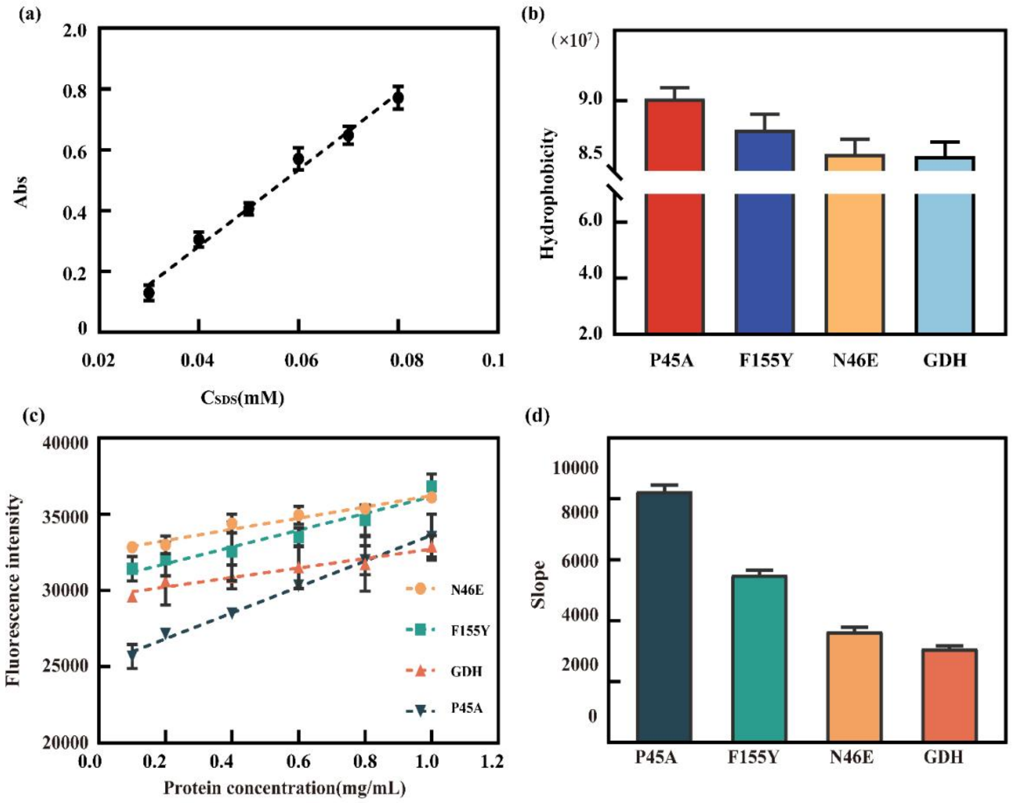

Supplementary Figure 10 Detection of GDH and its hydrophobicity using SDS binding and curcumin. (a): Determination of absorbance of different concentrations of SDS solution, the regression equation is y=12.589x +0.7796 (R^2 = 0.9934). (b): The hydrophobic value is measured by the SDS binding method. The SDS-binding capacity was determined as follows: SDS was added in 10 mL of a 1 mg/mL protein solution and adjusted to 0.07 mM. After being allowed to stand 30 min, SDS-protein mixtures were dialyzed against 25 volumes of 0.02 M phosphate buffer, pH 6.0, for 24 h. Ten milliliters of $CHCl_3$ was added to 0.5mL of inner dialyzates and mixed in a test tube. Then 2.5 mL of a 0.0024% methylene blue solution was added to the $CHCl_3$ layer. After being mixed in the test tube, the mixture was centrifuged at 2500 rpm. The absorbance of the SDS-methylene blue mixture in the lower layer was measured at 655 nm. SDS binding capacity (µg of SDS bound to 1 mg protein) indicates the measure of the hydrophobicity of protein. (c): Fitting curves of different protein concentrations under the action of curcumin. (d): The hydrophobic index of GDH mutants. Different concentrations stock solutions of was prepared in 50 mM Tris-HCl (pH 7.4). Curcumin was titrated against these different protein solutions under constant stirring. The excitation wavelength was 430 nm with the slit widths of 5 and 10 nm. The increase in fluorescence with the addition of protein at respective emission maxima was recorded.
